# Pupillary Pain Index Predicts Postoperative Pain but Not the Effect of Peripheral Regional Anaesthesia in Patients Undergoing Total Hip or Total Knee Arthroplasty: An Observational Study

**DOI:** 10.3390/medicina59050826

**Published:** 2023-04-23

**Authors:** Evgeniya Kornilov, Lena Gehlen, Dana Yacobi, Martin Soehle, Ana Kowark, Marcus Thudium

**Affiliations:** 1 Department of Anaesthesiology and Intensive Care Medicine, University Hospital Bonn, Venusberg Campus 1, 53127 Bonn, Germany; 2 Department of Anaesthesia, Rabin Medical Center, Beilinson Hospital, 39 Jabotinsky Street, Petach Tikva 4941492, Israel; 3 Department of Neurobiology, Weizmann Institute of Science, 234 Herzl Street, Rehovot 7610001, Israel

**Keywords:** pupillary pain index, nociception monitoring, pupillary dilation reflex, postoperative pain, fascia iliaca block, adductor canal block

## Abstract

*Background and Objectives*: The pupillary pain index (PPI) allows the evaluation of intraoperative nociception by measuring pupillary reaction after a localized electrical stimulus. It was the objective of this observational cohort study to investigate the pupillary pain index (PPI) as a method to evaluate the fascia iliaca block (FIB) or adductor canal block (ACB) sensory areas during general anaesthesia in orthopaedic patients with lower-extremity joint replacement surgery. *Materials and Methods*: Orthopaedic patients undergoing hip or knee arthroplasty were included. After anaesthesia induction, patients received an ultrasound-guided single-shot FIB or ACB with 30 mL and 20 mL of 0.375% ropivacaine, respectively. Anaesthesia was maintained with isoflurane or propofol/remifentanil. The first PPI measurements were performed after anaesthesia induction and before block insertion, the second at the end of surgery. Pupillometry scores were evaluated in the area of the femoral or saphenous nerve (target) and C3 dermatome (control). Primary outcomes were differences between PPIs before and after peripheral block insertion as well as the relationship between PPIs and postoperative pain scores; secondary outcomes were the relationship between PPIs and opioid requirements after surgery. *Results*: PPI decreased significantly from the first to the second measurement (4.17 ± 2.7 vs. 1.6 ± 1.2, *p* < 0.001 for target; 4.46 ± 2.7 vs. 2.17 ± 2.1, *p* < 0.001 for control). Control and target measurements did not show significant differences. A linear regression analysis showed that early postoperative pain scores could be predicted with intraoperative piritramide with improved prediction after adding PPI scores, PCA opioids and surgery type. Forty-eight-hour pain scores at rest and in movement were correlated with intraoperative piritramide and control PPI after the PNB in movement and with second-postoperative-day opioids and target PPI scores before block insertion, respectively. *Conclusions*: While the effect of an FIB and ACB could not be shown with PPI postoperative pain scores due to a large effect of opioids, perioperative PPI was shown to be associated with postoperative pain. These results suggest that preoperative PPI may be used to predict postoperative pain.

## 1. Introduction

The fascia iliaca block (FIB) and the adductor canal block (ACB) as parts of peripheral regional anaesthesia (PRA) have become standard of care in patients undergoing total hip (THR) or total knee replacement (TKR), respectively [1]. It has been shown that PRA in this cohort of patients decreases postoperative pain and opioid consumption and improves postoperative mobilization and rehabilitation [2]. Despite significant advantages, there are several factors which can influence the effectiveness of pain management by PRA. These factors include the ultrasound device itself as well as the technician’s expertise. Patient obesity, tissue oedema, muscle atrophy and anatomical variations can also deleteriously affect the outcome [3]. Furthermore, evaluating the analgesic efficacy of PRA under general anaesthesia can be challenging.

Videopupillometry is a novel method which uses the registration of the pupillary response to a standardized local electrical stimulus applied to the skin area of interest [4]. It can be used to predict analgesia depth before the actual surgical stimulus occurs. Tetanic stimuli of increasing intensity are delivered by a device and the pupillary response is registered in infrared light. The method utilizes the physiological principle of the pupillary dilation reflex (PDR) which is a pupil dilation in response to pain. In order to determine the extent of the pupil reaction, the pupillary pain index (PPI) is normalized to fit a range from 1 to 10 (from no pain to maximal pain) [5]. Recent studies have assessed the PPI for an evaluation of epidural anaesthesia and a thoracic paravertebral block in anaesthetized patients [6,7,8]. We assumed that videopupillometry may be a useful tool to test the efficacy of PRA during general anaesthesia. Therefore, we aimed to investigate the PPI in a prospective observational study in the setting of hip or knee arthroplasty. We hypothesized a relationship between the PPI after a peripheral nerve block and postoperative pain and opioid consumption.

## 2. Materials and Methods

### 2.1. Ethics

This prospective observational study was performed at the University of Bonn, Germany, between January and May 2019 and registered at the German clinical trials registry (DRKS00014304, 13 April 2018). Ethical approval for this study was provided by the ethics committee of the University of Bonn (no. 385/17, chairperson: Prof. Dr. K. Racké, date of approval: 20 March 2018).

### 2.2. Perioperative Management

Patients undergoing THR or TKR under general anaesthesia with PRA were included in this study after informed consent. Patients with recorded substance abuse, age <18 years and an ASA score >3 were excluded from the study.

In the operation room, an intravenous line was inserted, and patients were connected to the ASA standard monitoring and bispectral index monitoring (BIS, BIS Vista, Covidien Medtronic, Dublin, Ireland). Anaesthesia was induced with 0.2 mg fentanyl, 2 mg/kg propofol and 0.6 mg/kg rocuronium, followed by intubation and mechanical ventilation. For maintenance, a total intravenous anaesthesia with propofol and remifentanil or a balanced anaesthesia with isoflurane and remifentanil was used. The BIS was targeted between 40 and 60 as per standard hospital protocol. After intubation, patients received a peripheral nerve block. Patients with THR received an FIB. Most patients with TKR received an ACB although some TKR patients received an FIB to avoid interference with the surgical field.

For the FIB, the fascia iliaca was localized with ultrasound at the level of the inguinal crease. A quantity of 30 mL of ropivacaine 0.375% was injected beneath the fascia iliaca usually about 3 cm lateral to the femoral artery to reach a lateral and medial spread of the local anaesthetic.

For the ACB, the ultrasound transducer was positioned on the anteromedial side of the thigh so that the sartorius muscle could be seen directly above the femoral artery and proximal to the point where the artery descends through the adductor canal. Then, 20 mL of ropivacaine 0.375% was injected laterally to the artery at the location of the saphenous nerve.

Patients received intraoperative analgesia with remifentanil at a rate of 0.1–0.3 µg/kg/min. At the time of fascia closure, patients received a piritramide IV bolus according to the anaesthesiologist’s judgement. In addition, all patients received 1–2 g of metamizole intraoperatively.

After surgery, patients were extubated and transferred to the postanaesthesia care unit (PACU), where they were connected to a patient-controlled analgesia device (PCA). Patients were discharged from the PACU when they were fully awake, hemodynamically stable, with NRS ≤ 3.

Total-knee-replacement patients received PCAs with piritramide and THR patients received PCA with a tramadol–metamizole combination. Tramadol PCA patients received continuous tramadol (10 mg/h) as well as boluses (10 mg) with a maximal 4 h dose of 90 mg, while the piritramide PCA patients could receive 2 mg boluses every 8 min with maximal 4 h dose of 30 mg. In the orthopaedic ward, patients were interviewed about their current pain experiences using the numeric rating scale (NRS) at 24 and 48 h at rest and in movement during mobilization. The doses of opioids administered via PCA (24 h, 48 h, total) were also recorded.

### 2.3. Pupillometry Measurement

Electrodes of a pupillometry device (MAPstation, IDmed, Marseille, France) were applied on the skin area affected by regional anaesthesia (target measurement) and the C3 dermatome in the supraclavicular fossa (control measurement). Target site for the ACB was the dermatome of the saphenous nerve distal to the injection site, and for the FIB, the dermatome of the femoral nerve 10 cm distal to the inguinal crease. The target and the control PPI scores were assessed twice, first after anaesthesia induction before remifentanil was started and before block insertion, and second, at the time of skin suture.

A handheld infrared camera registered the pupil size automatically. Once the measurement process was started, an increasing electric current of up to 60 mA was applied to the electrodes in 10 mA steps. From the pupillary reaction, the PPI score was automatically generated and recorded.

### 2.4. Bias

Measurement bias: PPI measurements were performed by the same researcher and did not affect the decisions of the staff. NRS scores measurements and recordings of the opioid doses from PCA devices were performed by nursing staff as per routine protocol. Selection bias was decreased by enrolling study participants consecutively. General anaesthesia with the use of opioids can represent a potential confounder which cannot be ruled out completely. The positioning of the control site electrodes may have influenced the control measurements. In addition, unknown potential factors could have affected the findings presented here.

### 2.5. Sample-Size Calculation

Based on a previous pilot study [5], it was estimated that the mean PPI value would be about 6 after anaesthesia induction and before the peripheral block. Considering the difference between the mean values before and after the block to be at least 20%, with an assumed standard deviation of 2.2, a minimum of 29 patients had to be included in order to give the study a power of 80% with a type I error of 0.05. We expected a dropout rate of >30% due to technical difficulties and missing data. Therefore, we aimed for 40 patients. To compensate for known dropouts, n was increased to 44.

### 2.6. Outcomes

The primary outcomes were:

- The differences between PPIs before and after peripheral block insertion.
- The relationship between PPIs and NRS scales (PACU, 24 and 48 h, at rest and in movement).

The secondary outcome was the association between PPIs and opioid consumption from PCA devices (24, 48 h, total) in morphine equivalent.

### 2.7. Statistical Analysis

A statistical analysis was performed using SPSSv26 (IBM, Ehningen, Germany) and MATLAB R2020a (The MathWorks, Natick, USA) software. As PPI is an ordinal scale, nonparametric tests were used.

Morphine equivalents were calculated from PCA opioid doses as follows: piritramide PCA—1 mg of piritramide = 0.7 mg of morphine. Tramadol–metamizole PCA—1 mg of tramadol = 0.1 mg of morphine; metamizole was not taken into account [9].

Continuous variables are presented as mean ± SD. The Wilcoxon signed rank test was used to compare paired samples, the Mann–Whitney test was used to compare independent samples. A Spearman test was used to assess the correlation between target PPI after block and postoperative pain scores. Statistical significance was defined as *p* < 0.05 (two-sided).

### 2.8. Regression Analysis

To investigate whether PPI scores could predict postoperative pain levels, multiple linear regression models were constructed using a stepwise selection of *p* value < 0.2 and the models with the best adjusted R-squared value were reported. The dependent variable was the postoperative pain score (NRS PACU; NRS 24 h and 48 h, at rest and in movement). The independent variables were PPI scores from the first and second measurements in the target area and the control area, as well as surgery type (THR or TKR), PCA type (tramadol or piritramide) and perioperative opioids. For the NRS PACU, intraoperative piritramide was included as a covariate, and for the NRS 24 h and the NRS 48 h, intraoperative piritramide and total opioids on the first and second postoperative days were included, respectively. Considering the possible multicollinearity between the dependent variables, variance inflation factors (VIF) were also reported. Due to potential differences between surgery groups, separate regression models were also calculated for each group (THR and TKR). Additionally, simple linear regression analyses were performed for each dependent and independent variable for the entire study cohort, as well as for each group.

## 3. Results

**Figure 1:**
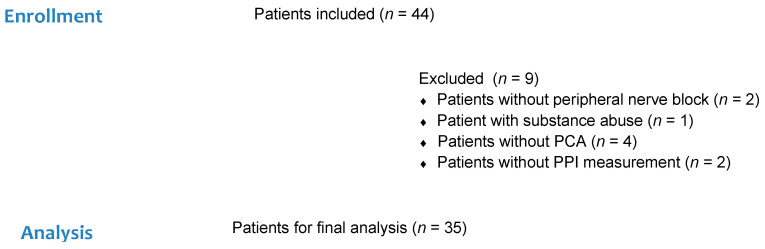
Flowchart of patient inclusion.

Forty-four (44) patients initially participated in the study, and 35 patients were included in the final analysis. The flowchart of patient inclusion is shown in Figure 1. Eighteen patients (51.4%) underwent THR and seventeen patients (48.6%) TKR. Perioperative patient characteristics are shown in Table 1. The comparisons of THR and TKR groups revealed an increased opioid consumption in THR patients after 24 h and 48 h with equal postoperative NRS scores, although NRS was slightly increased after 48 h in movement in THR patients (*p* = 0.05, supplementary material: Table S1).

Data are presented as number (%) and mean ± standard deviation; for age and body mass index—mean (range). The remifentanil dose ratio was calculated as a total remifentanil dose in mcg/surgery time in minutes. PCA—patient-controlled analgesia; NRS—numeric rating scale; PACU—postanaesthesia care unit; MED—morphine equivalent dose. For piritramide, MED was calculated as 1 mg of piritramide = 0.7 mg of morphine; for tramadol, 10 mg of tramadol = 1 mg of morphine.

### 3.1. Primary Outcomes

**Figure 2:**
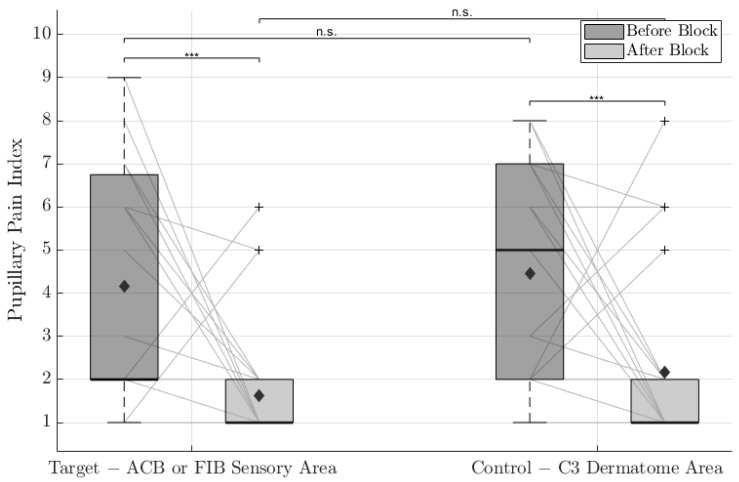
Pupillary pain index scores before and after a peripheral nerve block. Data are presented as boxplots. Paired measurements presented with additional grey lines corresponding to actual scores before and after block. Two boxplots (left side)—PPI from ACB or FIB sensory area. Two boxplots (right side)—control measurement from supraclavicular fossa. Black squared markers represent mean values. Significance: ***—*p* value < = 0.001; n.s.—nonsignificant. PPI—pupillary pain index. ACB—adductor canal block. FIB—fascia iliaca block.

PPI scores after the block were significantly lower than before the block both in the FIB and ACB sensory areas (4.17 ± 2.7 vs. 1.6 ± 1.2, *p* < 0.001) and the C3 dermatome area (4.46 ± 2.7 vs. 2.17 ± 2.1, *p* < 0.001). The comparison between control and target measurements did not show significant differences (Figure 2). We found a mild significant correlation between PPI after the block and the NRS 24 h at rest (r = 0.34, *p* = 0.046), as well as the NRS 48 h in movement (r = 0.41, *p* = 0.013). Other pain scores did not show significance in terms of PPI (NRS PACU: r = −0.15, *p* = 0.4; NRS 24 h in movement: r = 0.2, *p* = 0.3; NRS 48 h at rest: r = 0.31, *p* = 0.07).

**Figure 3:**
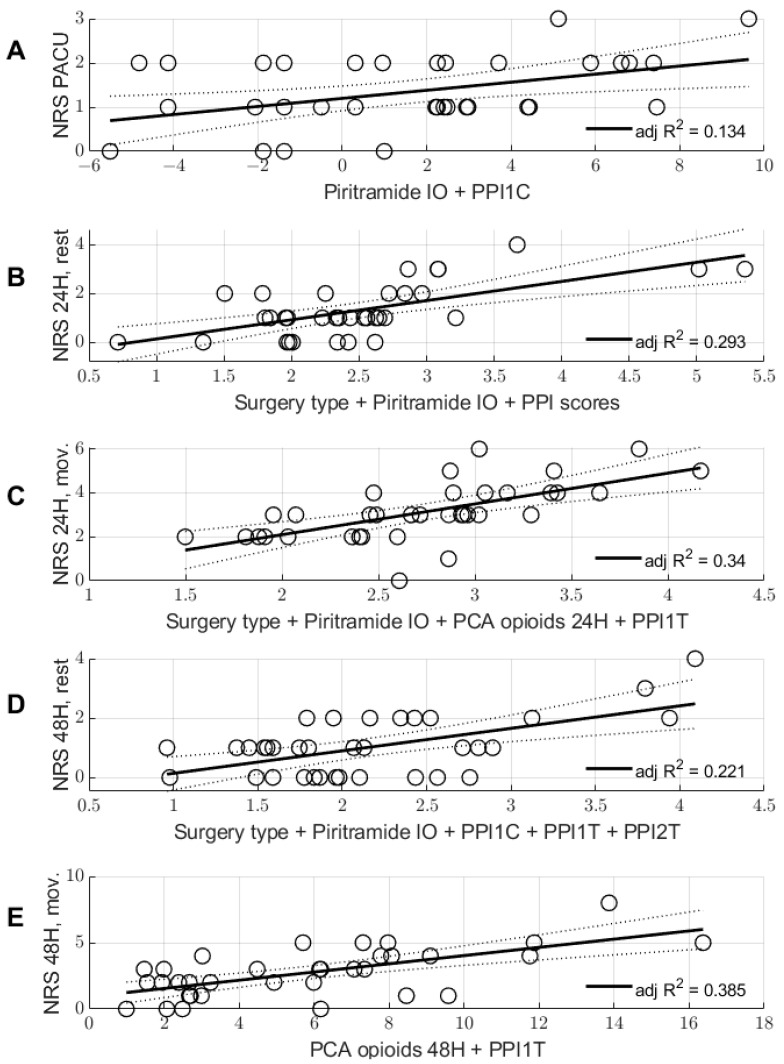
Linear models for pain scores ((**A**)—NRS PACU; (**B**)—NRS 24 h, rest; (**C**)—NRS 24 h, movement; (**D**)—NRS 48 h, rest; (**E**)—NRS 48 h, movement) prediction. For each model, the adjusted R squared is given (legends). Circles represent adjusted data; dotted lines—95% confidence bounds. NRS—numeric rating scale; PACU—postanaesthesia care unit; IO—intraoperative; PPI1C—control pupillary pain index before the block; PPI1T—target pupillary pain index before the block; PPI2T—target pupillary pain index after the block; PCA—patient-controlled analgesia.

Early postoperative scores (NRS PACU, NRS 24 h (at rest and in movement)) could be predicted with intraoperative piritramide (Table S2). Adding to the model the PPI scores, PCA opioids and surgery type could improve the prediction. Forty-eight-hour pain scores at rest were associated with intraoperative piritramide and control PPI after the PNB (Table S2). The model could be improved by adding other PPI scores and the surgery type. The NRS 48 h in movement correlated with the second-postoperative-day opioids and PPI scores in the FIB and ACB sensory areas before the block. The prediction models of postoperative pain scores are shown in Table 2. A graphical representation of each model is shown in Figure 3.

After splitting the models by surgery type, we could not predict the NRS PACU with the included independent variables. For the THR surgery, the NRS 24 h at rest was associated with intraoperative piritramide and the target PPI before PNB (adjusted R^2^ = 0.346, *p* = 0.03). The NRS 24 h in movement correlated with the same predictors as well as the first-postoperative-day opioids (adjusted R^2^ = 0.435, *p* = 0.011). The NRS 48 h in movement showed relationships with intraoperative piritramide and PPI scores (adjusted R^2^ = 0.723, *p* < 0.001). For the TKR surgery, the NRS 24 h in movement was associated with intraoperative piritramide (adjusted R^2^ = 0.213, *p* = 0.04). The NRS 48 h at rest connected to intraoperative piritramide and second-postoperative-day opioids (adjusted R^2^ = 0.292, *p* = 0.035); The NRS 48 h in movement correlated with the same predictors as well as with the PPI scores (adjusted R^2^ = 0.425, *p* = 0.028). The complete information concerning the regression analysis and prediction models for each group can be found in Tables S3 and S4, respectively.

### 3.2. Secondary Outcomes

No correlation could be observed between the PPIs and perioperative opioid consumption (Supplement 1: Table S5).

## 4. Discussion

Postoperative pain after joint arthroplasty is an important challenge for postoperative outcome. Postoperative mobilization can be facilitated via the use of peripheral regional anaesthesia, which can ultimately influence operative results. With the PPI, we aimed to assess the effectiveness of peripheral nerve blocks in THA and TKA surgeries. Although our data did not support an effect of the peripheral nerve block on PPI values, PPI values were associated with postoperative pain values.

PPI values alone were not associated with postoperative opioid consumption. However, the comparison between the THR and TKR groups revealed an increased opioid consumption in the THR group. It must be noted at the same time that all THR patients received an FIB while TKR patients received both an ACB and an FIB. While one might expect an increased opioid consumption in the TKR group, the opposite was the case [10,11]. We attribute this to the differences in the PCA pump type and differences in analgesic potency between the two opioids used, irrespective of the conversion into an MED.

### 4.1. Influence of Nerve Blocks and Opioids

The observed differences between PPI1 and PPI2 suggest that PPI can be considerably influenced by opioid administration. This finding is in line with previous reports from Sabourdin et al., who investigated the PPI during general anaesthesia with and without opioids [5]. In our patients, a clear effect of opioids, but not of the block, could be observed with the PPI. These results are in contrast to some previous studies that showed that the effect of a paravertebral block could be monitored by measuring the pupillary dilation reflex (PDR) with a tetanic stimulation of the dermatome of interest [7,8]. Isnardon et al. measured the PDR during general anaesthesia with a popliteal nerve block during a continuous infusion of remifentanil and showed that the PDR was lower compared to the nonblocked leg [12]. The assessment of thoracic epidural anaesthesia with pupillometry has also been previously described [6,7]. In this matter, our results diverge from previous findings for reasons that are yet unclear. However, one explanation could be that peripheral nerve blocks are not as effective as expected in patients with hip or knee replacement. Another reason for the variance could be that in our patients, more opioids were given, or patients were more sensitive to opioids than patients in the reports described above. The influence of opioids best explains our results especially considering the reduced PPI from PPI1 to PPI2 at both sites, indicating an increased generalized analgesia level most likely caused by opioids. This potential mechanism also corresponds with our finding that early postoperative pain scores are related to the perioperative piritramide application. Aissou et al. found that the PDR was correlated with pain scores in the immediate postoperative period after general surgery and could be a good tool to guide morphine administration in early postoperative period [13]. Pupil diameter fluctuations (pupillary unrest under ambient light) were shown to predict opioid analgesic efficacy [14]. We attribute the lack of variability between PPI2 at the target and at the block site to a large opioid effect rather than the negligible effect of the block. A mixed effect of the peripheral block and opioids may also explain the missing relationship between PPI2 at the target site and postoperative pain and opioid consumption.

### 4.2. Preoperative Nociception

Although the effect of the peripheral nerve block was not reflected in the PPI, PPI1 at the target site was related to postoperative pain on the second postoperative day in movement. Due to the active nature of the PPI, the method seems to be especially useful in predicting pain in postoperative mobilization. These results are in line with previous findings that reported the usefulness of a preoperative heat stimulus to predict postoperative pain in knee surgery [15]. A linear regression analysis showed that while postoperative NRS levels at 24 and 48 h were dependent on intra- and postoperative opioids, respectively, the addition of PPI values could improve the prediction model. This may suggest a role of preoperative nociception in the development of postoperative pain. Several reports propose such a connection. Martinez et al. reported on the relationship of primary hyperalgesia with postoperative pain in patients with TKA [16]. Lundblad et al. suggested that a low preoperative pain threshold may represent a central sensitization mechanism resulting in increased postoperative pain in TKA [11]. While Nielsen et al. utilized a preoperative electrical stimulus to predict post-caesarean section pain, Aasvang et al. could not predict postoperative pain outcomes in hernia pain with electrical stimulation [17,18]. This suggests that preoperative nociception measurement may be effective in some surgical areas. This phenomenon may be attributed to the underlying pathology and the sensitization of nociceptors in inflammation as well as central mechanisms resulting in oversensitive nociceptive neurons [19]. We confirmed that PPI may play a role in the prediction of postoperative pain in joint arthroplasty surgery. However, the relationships observed here and the effects on postoperative pain levels remain to be shown by larger studies with a longer follow-up time.

There are several limitations inherent in this study. TKA and THA patients represent different groups of patients. Additionally, in our cohort the group sizes receiving an ACB and FIB were different, and different nerve blocks were applied in the TKA group. A postoperative management with a tramadol mixture in THA patients as opposed to TKA patients with piritramide PCAs adds to the difficulty of comparing the two groups. Furthermore, we presented a relatively small cohort of patients with a relatively short follow-up period and therefore, results are of preliminary nature and should be interpreted in this context. We performed regional anaesthesia after the induction of general anaesthesia, although this is not ubiquitously practiced. Therefore, the results presented here may not be applicable in all circumstances.

## 5. Conclusions

Our results do not support the use of the PPI for the evaluation of an FIB and ACB when used in conjunction with opioids. However, the PPI may have a role in the prediction of postoperative pain. We suggest that the degree of postoperative pain may be predetermined even before surgical skin incision.

## Figures and Tables

**Table 1:** Perioperative patient characteristics.

Parameter	*N* = 35
Age, years	69.2 (47–90)
Body mass index	29 (19.4–46.9)
Surgery type, *n* (%):	
Total hip replacement	18 (51.4%)
Total knee replacement	17(48.6%)
Anaesthesia type, *n* (%):	
General anaesthesia	32 (91.4%)
Total intravenous anaesthesia	3 (8.6%)
Regional anaesthesia type, *n* (%):	
Fascia iliaca block	27 (77.1%)
Adductor canal block	8 (22.9%)
Anaesthesia time, min	211.7 ± 47.5
Surgery time, min	131.4 ± 95
Intraoperative opioids:	
Remifentanil dose ratio	7.3 ± 4.8
Fentanyl, mg	0.33 ± 0.13
Piritramide in MED, mg	4.48 ± 3.36
Postoperative opioids dosage:	
24-h PO opioids in MED, mg	21.17 ± 13.18
48-h PO opioids in MED, mg	28.25 ± 39.09
PCA type, n (%):	
Tramadol	16(45.7%)
Piritramide	19 (54.3%)
Postoperative NRS:	
NRS PACU	1.34 ± 0.8
NRS 24 h, rest	1.31 ± 1.08
NRS 24 h, movement	3.1 ± 1.3
NRS 48 h, rest	1.03 ± 1
NRS 48 h, movement	2.7 ± 1.8

**Table 2:** Prediction models of postoperative pain scores.

NRS PACU. Adjusted R Squared for the Model = 0.134, *p*-Value = 0.038		VIF
Piritramide perioperative in MED, mg	0.066 (0.012–0.121, *p* = 0.019)	1.01
Control PPI before block	−0.063 (−0.161–0.035, *p* = 0.199)	1.01
NRS 24 h at rest. Adjusted R squared for the model = 0.293, *p* value = 0.013		
Surgery type	0.626 (−0.067–1.318), *p* = 0.075	1.215
Piritramide perioperative in MED, mg	0.096 (0.02–0.173), *p* = 0.015	1.319
Control PPI before block	−0.126 (−0.315–0.063), *p* = 0.183	2.536
Target PPI before block	0.159 (−0.029–0.346), *p* = 0.094	2.555
Control PPI after block	−0.147 (−0.337–0.042), *p* = 0.123	1.554
Target PPI after block	0.394 (0.08–0.707), *p* = 0.016	1.486
NRS 24 h in movement. Adjusted R squared for the model = 0.34, *p* value = 0.02		
Surgery type	1.394 (0.499–2.289), *p* = 0.003	1.485
Piritramide perioperative, mg	0.114 (0.025–0.204), *p* = 0.014	1.333
PCA opioids 24 h	0.031 (−0.004–0.066), *p* = 0.082	1.539
Target PPI before block	0.095 (−0.049–0.238), *p* = 0.189	1.098
NRS 48 h at rest. Adjusted R squared for the model = 0.221, *p* value = 0.029		
Surgery type	0.666 (0.011–1.321), *p* = 0.046	1.187
Piritramide perioperative, mg	0.073 (0.002–0.143), *p* = 0.044	1.235
Control PPI before block	−0.191 (−0.364-−0.019), *p* = 0.031	2.304
Target PPI before block	0.151 (−0.024–0.325), *p* = 0.088	2.416
Target PPI after block	0.256 (0.001–0.511), *p* = 0.049	1.075
NRS 48 h in movement. Adjusted R squared for the model = 0.385, *p* value < 0.001		
Target PPI before block	0.309 (0.124–0.495), *p* = 0.002	1.032
PCA opioids 48 h	0.018 (0.005–0.03), *p* = 0.009	1.032

Prediction models of postoperative pain scores. Data are presented as unstandardized B coefficient (95% CI), *p* value. VIF—variance inflation factor; MED—morphine equivalent dose; PCA—patient-controlled analgesia; NRS—numeric rating scale; PACU—post anaesthesia care unit, PPI—pupillary pain index.

## Data Availability

Raw data have been submitted as a supplementary file (Supplement 2).

## Supplementary Materials

The following supporting information can be downloaded at: https://www.mdpi.com/article/10.3390/medicina59050826/s1, Table S1: Total hip replacement (THR) and total knee replacement (TKR) groups comparisons; Table S2: Simple regression analysis for independent variables of pain scores; Table S3: Prediction models of postoperative pain scores separately for total hip replacement and total knee replacement surgeries; Table S4: Simple regression analysis for independent variables of pain scores separately for total hip and total knee replacement surgeries; Table S5: Relationship between PPI indices and perioperative opioid consumption; Raw data.
