# Supplementary material for: Pupillary Pain Index Predicts Postoperative Pain but Not the Effect of Peripheral Regional Anaesthesia in Patients Undergoing Total Hip or Total Knee Arthroplasty: An Observational Study
Source: Medicina (Kaunas). 2023 Apr 23;59(5):826. doi: 10.3390/medicina59050826 (PMC10222659; doi:10.3390/medicina59050826)
Supplement: Supplementary file 1 [file medicina-59-00826-s001.zip › medicina-2229233-supplementary.pdf]

Table S1. Total hip replacement (THR) and total knee replacement (TKR) groups comparisons.

Parameter	THR group, N = 18	TKR group, N = 17	P value
Age, years	63.11 ± 14.76	71.94 ± 9.7	0.11
Body mass index	28.26 ± 7.73	30.57 ± 4.8	0.08
**Anesthesia type, n(%):**			
General anesthesia	18(100%)	14(82.4%)	0.06
Total intravenous anesthesia	0(0%)	3(17.6%)	
**Regional anesthesia type, n(%):**			
Fascia iliaca block	18(100%)	9(52.9%)	<0.001
Adductor canal block	0(0%)	8(47.1)	
Anesthesia time, min	225.11 ± 51.25	201.76 ± 46	0.17
Surgery time, min	149.94 ± 127.92	111 ± 36.97	0.43
**Intraoperative opioids dosage:**			
Remifentanil, mcg	8.52 ± 5.33	6.25 ± 4.62	0.21
Fentanyl, mg	0.36 ± 0.11	0.36 ± 0.16	0.88
Piritramide in MED, mg	6.3 ± 6.86	3.25 ± 2.86	0.13
**Postoperative opioids dosage:**			
24-hours PO opioids in MED, mg	35.53 ± 46.04	15.47 ± 13.09	0.01
48-hours PO opioids in MED, mg	44.1 ± 52.36	16.69 ± 28.27	0.03
**PCA type, n(%):**			
Tramadol	12(66.7%)	4(23.5%)	0.01
Piritramide	6(33.3%)	13(76.5%)	0.01
**Postoperative NRS:**			
NRS PACU	1.44 ± 0.7	1.41 ± 0.87	0.97
NRS 24h, rest	1.61 ± 1.42	1.41 ± 1.46	0.69
NRS 24h, movement	3.56 ± 2.01	3.06 ± 1.39	0.83
NRS 48h, rest	1.67 ± 1.91	0.94 ± 1.03	0.24
NRS 48h, movement	3.17 ± 1.58	2.12 ± 1.41	0.05

Data is presented as number (%), and mean ± standard deviation; for age and body mass index – mean (range). Remifentanil dose ratio calculated as a total remifentanil dose in mcg/surgery time in minutes. PCA – patient controlled analgesia; NRS – Numeric rating scale; PACU – post anaesthesia care unit; MED – morphine equivalent dose. For piritramide MED is calculated as 1 mg of piritramide = 0.7 mg of morphine; for tramadol – 10 mg of tramadol = 1 mg of morphine. P value for continuous variables – Mann-Whitney test; for categorical variables – Chi-square test.

Table S2. Simple regression analysis for independent variables of pain scores.

Simple regression	Unstandardized B coefficient (95% CI), p value	Adjusted r squared
**NRS PACU**		
Surgery type	-0.324(-0.872-0.225), p = 0.239	0.013
PCA type	0.059(-0.503-0.621), p = 0.832	-0.029
Piritramide perioperative in MED, mg	0.063(0.008-0.118), p = 0.026	0.115
Control PPI before block	-0.051(-0.155-0.053), p = 0.327	0
Target PPI before block	-0.008(-0.113-0.096), p = 0.874	-0.03
Control PPI after block	-0.014(-0.149-0.122), p = 0.837	-0.029
Target PPI after block	-0.03(-0.259-0.2), p = 0.795	-0.028
**NRS 24-hour at rest**		
Surgery type	0.19(-0.561-0.94), p = 0.611	-0.022
PCA type	-0.118(-0.873-0.636), p = 0.751	-0.027
Piritramide perioperative in MED, mg	0.098(0.026-0.17), p = 0.009	0.165
PCA opioids 24-hour	0.015(-0.013-0.044), p = 0.29	0.005
Control PPI before block	0.053(-0.088-0.194), p = 0.45	-0.012
Target PPI before block	0.108(-0.027-0.243), p = 0.114	0.046
Control PPI after block	-0.066(-0.247-0.115), p = 0.461	-0.013
Target PPI after block	0.193(-0.107-0.494), p = 0.2	0.02
**NRS 24-hour in movement**		
Surgery type	0.637(-0.249-1.523), p = 0.153	0.032
PCA type	-0.263(-1.176-0.65), p = 0.561	-0.02
Piritramide perioperative in MED, mg	0.109(0.02-0.197), p = 0.018	0.133
PCA opioids 24-hour	0.017(-0.017-0.052), p = 0.314	0.001
Control PPI before block	0.097(-0.072-0.266), p = 0.253	0.01
Target PPI before block	0.156(-0.006-0.317), p = 0.058	0.078
Control PPI after block	-0.039(-0.26-0.182), p = 0.72	-0.026
Target PPI after block	0.055(-0.319-0.429), p = 0.768	-0.028
**NRS 48-hour at rest**		
Surgery type	0.402(-0.271-1.075), p = 0.233	0.014
PCA type	-0.053(-0.742-0.637), p = 0.878	-0.03
Piritramide perioperative in MED, mg	0.052(-0.018-0.122), p = 0.143	0.036
PCA opioids 24-hour	0(-0.027-0.026), p = 0.978	-0.03
PCA opioids 48-hour	0.005(-0.004-0.013), p = 0.294	0.004
Control PPI before block	-0.043(-0.172-0.086), p = 0.505	-0.016

Target PPI before block	0.043(-0.084-0.171), p = 0.496	-0.016
Control PPI after block	0.126(-0.034-0.287), p = 0.119	0.044
Target PPI after block	0.16(-0.115-0.436), p = 0.245	0.012
**NRS 48-hour in movement**		
Surgery type	-0.703(-1.941-0.535), p = 0.257	0.01
PCA type	0.181(-1.084-1.446), p = 0.773	-0.028
Piritramide perioperative in MED, mg	0.119(-0.008-0.246), p = 0.065	0.072
PCA opioids 24-hour	0.031(-0.017-0.078), p = 0.197	0.021
PCA opioids 48-hour	0.021(0.007-0.036), p = 0.005	0.188
Control PPI before block	0.174(-0.057-0.405), p = 0.134	0.038
Target PPI before block	0.353(0.154-0.553), p = 0.001	0.26
Control PPI after block	0.072(-0.233-0.377), p = 0.634	-0.023
Target PPI after block	0.255(-0.254-0.764), p = 0.316	0.001

Data presented as unstandardized B coefficient (95% CI), p value. MED – morphine equivalent dose; PCA – patient controlled analgesia; NRS – Numeric rating scale; PACU – post anesthesia care unit, PPI – pupillary pain index.

Regression models for THR and TKR separately.

Table S3: Prediction models of postoperative pain scores separately for total hip replacement and total knee replacement surgeries.

**Total hip replacement surgery, N = 18**		
**NRS PACU. Adjusted r squared for the model = 0.032, p-value = 0.038**		VIF
Piritramide perioperative in MER, mg	0.047(-0.033-0.128), p = 0.229	1
**NRS 24-hour at rest. Adjusted r squared for the model = 0.346, p value = 0.03**		
Piritramide perioperative, mg	0.104(-0.004-0.212), p = 0.058	1.058
Control PPI after block	-0.21(-0.547-0.128), p = 0.204	1.315
Target PPI after block	0.444(0.083-0.804), p = 0.019	1.253
**NRS 24-hour in movement. Adjusted r squared for the model = 0.435, p value = 0.011**		
Piritramide perioperative, mg	0.076(-0.041-0.193), p = 0.185	1.343
PCA opioids 24-hour	0.023(-0.013-0.059), p = 0.193	1.198
Target PPI before block	0.181(-0.006-0.368), p = 0.057	1.178
**NRS 48-hour at rest. Adjusted r squared for the model = 0.051, p value = 0.186**		
Target PPI after block	0.139(-0.074-0.352), p = 0.186	1

**NRS 48-hour in movement. Adjusted r squared for the model = 0.723, p value <0.001**		
Piritramide perioperative, mg	-0.086(-0.216-0.044), p = 0.175	1.194
Control PPI before block	-1.093(-1.851--0.336), p = 0.008	14.287
Target PPI before block	1.699(0.919-2.479), p = 0	14.73
Control PPI after block	1.02(0.22-1.821), p = 0.016	5.778
**Total knee replacement surgery, N = 17**		
**NRS PACU. Adjusted r squared for the model = 0.073, p-value = 0.154**		VIF
Piritramide perioperative in MER, mg	0.068(-0.029-0.165), p = 0.154	1
**NRS 24-hour at rest. Adjusted r squared for the model = 0.282, p value = 0.151**		
PCA type	0.755(-0.646-2.156), p = 0.257	1.487
PCA opioids 24-hour	0.053(-0.031-0.137), p = 0.191	1.826
Control PPI before block	-0.457(-0.925-0.012), p = 0.055	6.719
Target PPI before block	0.532(0.14-0.924), p = 0.013	5.027
Control PPI after block	0.603(-1.055--0.151), p = 0.014	5.076
Target PPI after block	0.876(0.002-1.75), p = 0.05	3.05
**NRS 24-hour in movement. Adjusted r squared for the model = 0.213, p value = 0.036**		
Piritramide perioperative, mg	0.156(0.012-0.299), p = 0.036	1
**NRS 48-hour at rest. Adjusted r squared for the model = 0.292, p value = 0.035**		
Piritramide perioperative, mg	0.091(-0.037-0.22), p = 0.15	1.135
PCA opioids 48-hour	0.021(-0.004-0.045), p = 0.089	1.135
**NRS 48-hour in movement. Adjusted r squared for the model = 0.425, p value = 0.028**		
Piritramide perioperative, mg	0.099(-0.059-0.256), p = 0.198	1.161
PCA opioids 48-hour	0.021(-0.009-0.052), p = 0.148	1.178
Target PPI before block	0.222(-0.03-0.474), p = 0.079	1.119
Target PPI after block	0.717(-0.004-1.439), p = 0.051	1.12

Data presented as unstandardized B coefficient (95% CI), p value. VIF – variance inflation factor; MED – morphine equivalent dose; PCA – patient controlled analgesia; NRS – Numeric rating scale; PACU – post anesthesia care unit, PPI – pupillary pain index.

Table S4: Simple regression analysis for independent variables of pain scores separately for total hip and total knee replacement surgeries.

	Total hip replacement surgery, N = 18		Total knee replacement surgery, N = 17	
Simple regression	Unstandardized B coefficient (95% CI), p value	Adjusted r squared	Unstandardized B coefficient (95% CI), p value	Adjusted r squared
**NRS PACU**				
PCA type	0.25(-0.511-1.011), p = 0.496	-0.031	-0.558(-1.626-0.51), p = 0.283	0.015
Piritramide perioperative in MER, mg	0.047(-0.033-0.128), p = 0.229	0.032	0.068(-0.029-0.165), p = 0.154	0.073
Control PPI before block	-0.017(-0.158-0.125), p = 0.804	-0.058	-0.074(-0.244-0.096), p = 0.37	-0.009
Target PPI before block	-0.009(-0.152-0.135), p = 0.9	-0.061	0.004(-0.165-0.173), p = 0.956	-0.066
Control PPI after block	0(-0.235-0.235), p = 1	-0.063	0.002(-0.192-0.197), p = 0.98	-0.067
Target PPI after block	-0.028(-0.285-0.229), p = 0.822	-0.059	-0.047(-0.531-0.436), p = 0.837	-0.064
**NRS 24-hour at rest**				
PCA type	0.083(-1.133-1.3), p = 0.886	-0.061	-0.212(-1.546-1.123), p = 0.74	-0.059
Piritramide perioperative in MER, mg	0.109(-0.01-0.229), p = 0.069	0.141	0.129(0.026-0.232), p = 0.018	0.275
PCA opioids 24-hour	0.027(-0.014-0.068), p = 0.178	0.055	0.026(-0.045-0.097), p = 0.453	-0.026
Control PPI before block	0.093(-0.124-0.311), p = 0.377	-0.01	0.009(-0.202-0.219), p = 0.932	-0.066
Target PPI before block	0.125(-0.092-0.341), p = 0.239	0.028	0.089(-0.109-0.286), p = 0.355	-0.006
Control PPI after block	-0.095(-0.463-0.272), p = 0.59	-0.043	-0.071(-0.302-0.16), p = 0.521	-0.037
Target PPI after block	0.315(-0.055-0.685), p = 0.09	0.117	-0.069(-0.652-0.513), p = 0.803	-0.062
**NRS 24-hour in movement**				
PCA type	0.25(-1-1.5), p = 0.677	-0.051	-0.288(-2.068-1.491), p = 0.735	-0.058
Piritramide perioperative in MER, mg	0.145(0.032-0.258), p = 0.015	0.273	0.156(0.012-0.299), p = 0.036	0.213
PCA opioids 24-hour	0.041(0.001-0.08), p = 0.045	0.18	0.052(-0.04-0.144), p = 0.248	0.027

Control PPI before block	0.19(-0.017-0.397), p = 0.069	0.141	-0.013(-0.294-0.268), p = 0.921	-0.066
Target PPI before block	0.256(0.066-0.446), p = 0.011	0.296	0.047(-0.223-0.318), p = 0.715	-0.057
Control PPI after block	-0.131(-0.508-0.245), p = 0.47	-0.027	-0.047(-0.358-0.264), p = 0.752	-0.059
Target PPI after block	0.111(-0.304-0.526), p = 0.578	-0.042	-0.044(-0.822-0.734), p = 0.906	-0.066
**NRS 48-hour at rest**				
PCA type	0.25(-0.412-0.912), p = 0.435	-0.022	0.019(-1.556-1.594), p = 0.98	-0.067
Piritramide perioperative in MER, mg	0.021(-0.052-0.093), p = 0.554	-0.039	0.129(0-0.258), p = 0.05	0.182
PCA opioids 24-hour	0.005(-0.019-0.029), p = 0.637	-0.047	0.029(-0.055-0.112), p = 0.473	-0.029
PCA opioids 48-hour	0.002(-0.005-0.009), p = 0.538	-0.037	0.027(0.003-0.051), p = 0.03	0.23
Control PPI before block	-0.003(-0.127-0.121), p = 0.962	-0.062	-0.096(-0.338-0.146), p = 0.413	-0.018
Target PPI before block	-0.003(-0.128-0.123), p = 0.962	-0.062	0.069(-0.168-0.305), p = 0.544	-0.04
Control PPI after block	0.031(-0.174-0.236), p = 0.753	-0.056	0.146(-0.117-0.41), p = 0.255	0.025
Target PPI after block	0.139(-0.074-0.352), p = 0.186	0.051	0.226(-0.449-0.901), p = 0.486	-0.032
**NRS 48-hour in movement**				
PCA type	-0.417(-2.507-1.674), p = 0.678	-0.051	0.192(-1.889-2.274), p = 0.847	-0.064
Piritramide perioperative in MER, mg	0.059(-0.167-0.286), p = 0.587	-0.043	0.151(-0.025-0.327), p = 0.088	0.127
PCA opioids 24-hour	0.021(-0.053-0.096), p = 0.552	-0.039	0.027(-0.085-0.138), p = 0.617	-0.048
PCA opioids 48-hour	0.017(-0.003-0.037), p = 0.098	0.109	0.034(0.003-0.066), p = 0.035	0.213
Control PPI before block	0.376(0.046-0.706), p = 0.028	0.222	0.006(-0.322-0.334), p = 0.968	-0.067
Target PPI before block	0.571(0.324-0.818), p = 0	0.575	0.198(-0.1-0.496), p = 0.177	0.059
Control PPI after block	-0.273(-0.897-0.351), p = 0.367	-0.008	0.274(-0.057-0.606), p = 0.098	0.117
Target PPI after block	0.093(-0.606-0.792), p = 0.782	-0.057	0.588(-0.261-1.436), p = 0.161	0.069

Data presented as unstandardized B coefficient (95% CI), p value. MED – morphine equivalent dose; PCA – patient controlled analgesia; NRS – Numeric rating scale; PACU – post anesthesia care unit, PPI – pupillary pain index.

Regression models.

Dependent parameters: NRS PACU, NRS 24 and 48 hours at rest and in movement (5 parameters).

For NRS PACU:

Included parameters in the model: type of surgery; type of PCA (tramadol or piritramide); intraoperative piritramide in MED; target PPI before and after block; control PPI before and after block.

For NRS24:

Included parameters in the model: type of surgery; type of PCA (tramadol or piritramide); intraoperative piritramide in MED; PCA opioids 24H postoperative; target PPI before and after block; control PPI before and after block.

For NRS48:

Included parameters in the model: type of surgery; type of PCA (tramadol or piritramide); intraoperative piritramide in MED; PCA opioids 24H postoperative; PCA opioids 48H postoperative; target PPI before and after block; control PPI before and after block.

Table S5: Relationship between PPI indices and perioperative opioid consumption

	Intraoperative piritramide in MED	PCA opioids 24-hour	PCA opioids 48-hour
Control PPI before the block	r = 0.096, p value = 0.6	r = -0.13, p value = 0.5	r = -0.12, p value = 0.5
Target PPI before the block	r = 0.23, p value = 0.2	r = -0.11, p value = 0.5	r = 0.007, p value = 1
Control PPI after the block	r = -0.23, p value = 0.2	r = 0.19, p value = 0.3	r = 0.15, p value = 0.4
Target PPI after the block	r = 0.001, p value = 1	r = 0.19, p value = 0.3	r = 0.19, p value = 0.3

. PPI – pupillary pain index; PCA – patient controlled analgesia, MED – morphine equivalent dose. R coefficients are from Spearman correlation test.
